# Disaster preparedness among Thai elderly emergency department patients: a survey of patients’ perspective

**DOI:** 10.1186/s12873-019-0269-7

**Published:** 2019-10-23

**Authors:** Jiraporn Sri-on, Alissara Vanichkulbodee, Natchapon Sinsuwan, Rapeeporn Rojsaengroeng, Anucha Kamsom, Shan Woo Liu

**Affiliations:** 1 The Department of Emergency Medicine, Vajira Hospital, Navamindradhiraj University, Bangkok, Thailand; 2 The Department of Biostatistic, Vajira Hospital, Navamindradhiraj University, Bangkok, Thailand; 3 The Department of Emergency Medicine, Massachusetts General Hospital, Harvard Medical School, Boston, MA USA

**Keywords:** Disaster preparedness, Elderly, Emergency department

## Abstract

**Background:**

In disaster situations, the elderly are considered to be a particularly vulnerable population. Preparedness is the key to reduce post-disaster damage. There is limited research in middle-income countries on how well elderly emergency department (ED) patients are prepared for disaster situations. The objective of this study was to determine the attitudes and behavior of elderly ED patients toward disaster preparedness.

**Methods:**

This study was a cross-sectional face-to-face survey at one urban teaching hospital in Bangkok, Thailand between August 1st and September 30th, 2016. Patients aged 60 and older who presented to the ED were included to this study. We excluded patients who had severe dementia [defined as Short Portable Mental State Questionnaires (SPMSQ) > 8], were unable to speak Thai, had severe trauma and/or needed immediate resuscitation. The survey instruction was adapted from previous disaster surveys. This study was approved by the Vajira Institutional Review Board (IRB).

**Results:**

A total of 243 patients were enrolled. Most of them were female [154 patients (63.4%)]. The median age was 72 [Interquartile range (IQR) 66–81] years and the most common underlying diseases were hypertension [148 patients (60.9%)] and diabetes [108 patients (44.4%)]. The majority of patients [172 patients (72.4%)] reported that they had had some teaching about disaster knowledge from a healthcare provider and had experienced a disaster [138 patients (56.8%)]. While 175/197 (81.8%) patients who had underlying diseases reported that they had a medication supply for disaster situations, only 61 (25.1%) patients had an emergency toolbox for disasters. Most patients (159, 65.4%) did not know the emergency telephone number, and 133 (54.7%) patients reported transportation limitations.

**Conclusions:**

While most Thai elderly ED patients reported having a medication supply for disaster situations, many lacked comprehensive plans for a disaster situation. Work needs to be done to improve the quality of preparedness in disaster situations among elderly patients. Future research should focus on preparedness knowledge regarding evacuation, and shelter/residence for older patients.

## Background

In disaster situations, the elderly are considered to be a particularly vulnerable population [1, 2]. Older adults are usually more severely injured, have prolonged hospital length of stay, lower physical quality of life and psychological well-being, are slower to recover, and have a higher death rate compared with the younger aged group in disaster situations [3–7].

Preparedness is the key to reducing post-disaster difficulty. Preparedness among community dwelling patients includes disaster awareness, understanding of the disaster, as well as accepting the consequences of ignoring safety instructions, which may lead to injury, post-traumatic stress disorder (PTSD) and death [8–10].

One study in an Italian emergency department (ED) stated that only 45% [11] of EDs had a program for disaster preparedness. A study in the US [12], from North Carolina found 53% of emergency patients did not have a disaster plan and 46% had stored food and drinks for 3 days. Morin VM, et al. [2] conducted a household survey in Philippines, a country often affected by natural disasters, and found that less than 1% of adults were prepared for disaster situations.

Thailand is a middle-income country with a rapidly aging society. In 2020, one third of the Thai population will be older than 60 years [13]. Several disasters have occurred in recent years in Thailand, e.g. the Tsunami in 2004 and Bangkok flooding in 2011. After the Tsunami in 2004, Guerena-Burgueno F et al. [14] showed that the military and hospitals responded well to the disaster. The 2011 flood in Thailand was the worst flooding in terms of people affected. It impacted 1,886,000 households, destroyed 19,000 homes and affected 2.5 million people [15]. While most of the Thai disaster studies only focus on the disaster response phase and hospital preparedness [14, 16, 17], there has been no study of disaster preparedness among older adults in a middle-income country and how well elderly ED patients are prepared for disaster situations. The objective of this study was to determine the attitudes and behaviors of elderly ED patients for disaster preparedness.

## Methods

This study was a cross-sectional face-to-face survey study at one urban teaching hospital in Bangkok, Thailand. There are approximately 50,000 annual ED visits; 30 % of them are among those older than 60 years. This study was approved by our hospital’s institutional review board. We obtained a written informed consent from study participants.

### Participants

This study was a descriptive cross-sectional survey. We surveyed a convenience sample of patients 7 days a week between 8.00 am. and 12.00 am.(16 h/day). We included patients aged 60 and older who presented to the ED between August 1st and September 30th, 2016. We excluded patients who had severe dementia which was determined by using the short portable mental status questionnaire (SPMSQ) and had a score > 8, were unable to speak Thai, were totally blind, deaf, aphasic and had severe trauma and/or needed immediate resuscitation.

### Survey development process

The survey was created through item generation, construction, pilot testing and clarification. Firstly, items generation and survey construction: The survey was adapted from studies by Alrousan TM et al. [18] and Daugherty JD et al. [19]. Three attending emergency physicians (EPs) who were experts in the disaster field performed a focus group to clarify all survey questions. The survey instrument consisted of a 22-item questionnaire with a true-false choice and Likert scale response format. The survey contained 7 categories: baseline demographic, knowledge about disasters, preparedness in disaster situations, communication in disaster situations, community preparedness in disaster situations, experiences in disaster situations, and family support in disaster situations (Survey questions are presented in supplement 1).

Secondly, pilot testing and clarification: The survey was piloted by a group of 10 healthcare providers who worked in the hospital but were not physicians or nurses. We asked 10 healthcare providers to do a questionnaire and clarify the meaning of each questionnaire item. The approximate time to finish the survey in our pilot group was 10 min.

### Definitions

Community program was defined as any programs (basic and advanced) about disaster preparedness for community-dwelling populations.

Emergency tool box was defined as a basic tool kit which included the following items: water and food supply at least 3 days, flashlight, first aid kit, garbage bag, battery power, whistle to signal for help, manual can opener for food, dust mask, local map and cell phone. Additional items for elderly patients included a minimum 3 days supply of medications, hearing aids, glasses, information about medical devices such as walker, oxygen supply and identification (ID) band.

### Survey administration

Two research assistants (RAs) who were blind to the study hypothesis performed the survey data collection. Research assistants had 2 h of training to clarify data collection and the enrollment process. RAs approached patients to consent them. Patients who could read administered the survey items by themselves; RAs helped clarify terms and the meaning of questions. For those who had reading problems, RAs asked the patients the survey questions.

### Statistical analysis

Data were analyzed by using STATA version 15.0. We presented categorical data as percentage. Analysis of disaster preparedness indicators were compared between young older adults group (aged 60–74 years) and old older adults group (aged 75 and older; age related physiological and functional decline). Comparisons between categorical data were done using Chi-square or Fisher’s exact test where appropriate. We A *p*-value of 0.05 was considered significant.

## Results

**Fig. 1:**
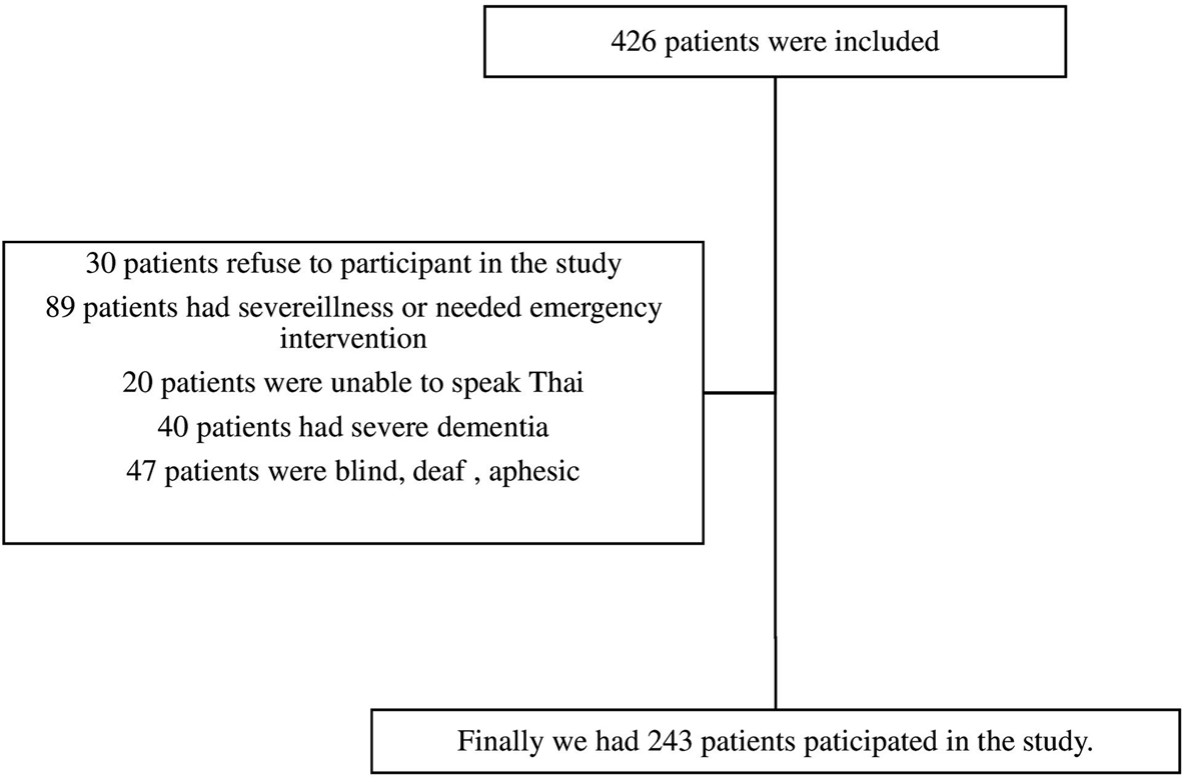
Subject enrollment

We surveyed 243 patients. Details of enrollment are presented in Fig. 1. Most of them were female [154 patients (63.4%)]. The median age was 72 [Interquartile range (IQR) 66–81] years and the most common underlying diseases were hypertension [148 patients (60.9%)] and diabetes [108 patients (44.4%)]. About half of the elderly ED patients [137 patients (56.4%)] were independent with basic activities of daily living (Table 1).

**Table 1:** Baseline Characteristics

Variables	*N* = 243(%)
Age, median (IQR)	72 (66–81)
SPMSQ Score, median (IQR)	4 (2–6)
Gender	
Female	154 (63.4)
Professional	
No work	197 (81.1)
Retired government employee	19 (7.8)
Housemaid	8 (3.3)
Business	19 (7.8)
Had monthly income	
Yes	44 (18.1)
No	199 (81.9)
Median income (IQR) Thai baht	10,000 (21,500)
Educations	
Primary school	140 (57.6)
Secondary School	55 (22.6)
College or Higher	25 (10.2)
Uneducated	23 (9.5)
Underlying Diseases	
Hypertension	148 (60.9)
Diabetes	108 (44.4)
Cardiovascular disease	56 (23.1)
Dyslipidemia	50 (20.6)
Chronic kidney disease	31 (12.8)
Chronic obstructive pulmonary disease or asthma	14 (5.8)
Cancer	11 (4.5)
Cerebrovascular disease	10 (4.1)
Functional status	
Doing basic activities of daily living	137 (56.4)
Used cane or walker to ambulate	85 (34.9)
Needed help to ambulate	18 (7.4)
Bed ridden	3 (1.2)

### Knowledge about disaster (Table 2)

Two-thirds of patients [162 patients (66.8%)] reported that they had good knowledge about disaster preparedness (points 4 and 5 on Likert scale). Two hundred and thirteen (87%) patients reported having had healthcare providers provide knowledge about disasters (point 4 and 5 on Likert scale). One hundred and sixty seven (69%) patients state that they knew the risk for disaster in the community (point 4 and 5 on Likert scale).

**Table 2:** Preparedness knowledge for disaster

Questions					
	Most 5 N(%)	4 N(%)	3 N(%)	2 N(%)	Least 1 N(%)
1. Have you ever had knowledge about disaster preparedness for example flood, cyclone, emerging infectious diseases?	103 (42)	59(24)	52(21)	23(10)	6(3)
2. Have you ever participated in a disaster preparedness course?	177 (73)	19(8)	29(12)	15(6)	3(1)
3. Have you ever had a healthcare provider provide knowledge about disasters?	176 (72)	37(15)	16(7)	12(5)	2(1)
4. Do you know the risk of disaster in your community?	100 (41)	67(28)	51(21)	23(10)	2(1)
5. Have you ever had an emergency plan for disaster situations?	139(57)	55(23)	28(12)	18(7)	3(1)

### Preparedness in disaster situations (Table 3)

In this survey 48(19.8%) patients reported knowing the specific location of emergency shelters. One hundred and ninety seven (81.1%) patients had underlying diseases that required long-term medications. 175/197(81.8%) patients who had underlying diseases reported that they had a medication supply for disaster situations of at least 3 weeks. The survey showed only 61 (25.1%) patients had an emergency toolbox for disasters.

**Table 3:** Disaster preparedness indicator

Variables	60–74 Yrs *N* = 141 (%)	> 74 Yrs *N* = 102 (%)	*P value*
Known specific location of shelter in emergency situation	30 (21.3)	18 (17.7)	0.48
Had emergency tool box	37 (26.2)	24 (23.5)	0.63
Had medications supply in disaster situation	101/115(87.8)	74/82 (90.2)	0.59
Limited mobility when need to transfer	61 (43.3)	72 (70.6)	< 0.001
Used natural gas in residence	133 (94.3)	94 (92.2)	0.50
Had medical devices with electronic supply	8 (5.7)	6 (5.9)	0.95
Had power cut off system or knew how to turn off abnormal electrical supply	14 (9.9)	6 (5.9)	0.26
Knew emergency telephone number	59 (41.8)	25 (24.5)	0.005
Knew how to contact organization for help in emergency situation	85 (60.3)	49 (48.0)	0.06
Had telephone for emergency call	122 (86.5)	59 (57.8)	< 0.001

One hundred and fifty nine (65.4%) patients did not know the emergency telephone number for ambulance. When we compared the young older adults group (aged 60–74 years) with old older adults group (aged 75 and older; age related physiological and functional decline) we found that the old older adults group knew fewer emergency phone numbers than the young older adults group [25(24.5%) versus (vs.) 59(41.8%) *p* value 0.005], and the old older adults group had fewer telephones for emergency calls than young older adults group [59(57.8%) vs. 122(86.5%) p value < 0.001]. Likewise, 133 (54.7%) patients in old older adults group reported having more transportation limitations than young older adults group [72(70.6%) vs. 61(43.3%) p value < 0.001].

### Community and family preparedness (Fig. 2)

Thirty-two (13.2%) elderly ED patients had participated in a community disaster preparation program. One hundred and twelve patients (46.09%)] thought that their community had a plan for disaster preparedness. One hundred and thirty eight (56.8%) had experienced a disaster event, especially the Bangkok flood in 2011. Interestingly, most of the elderly ED patients [226 patients (93%)] had a household member who could help in disaster situations.

**Fig. 2:**
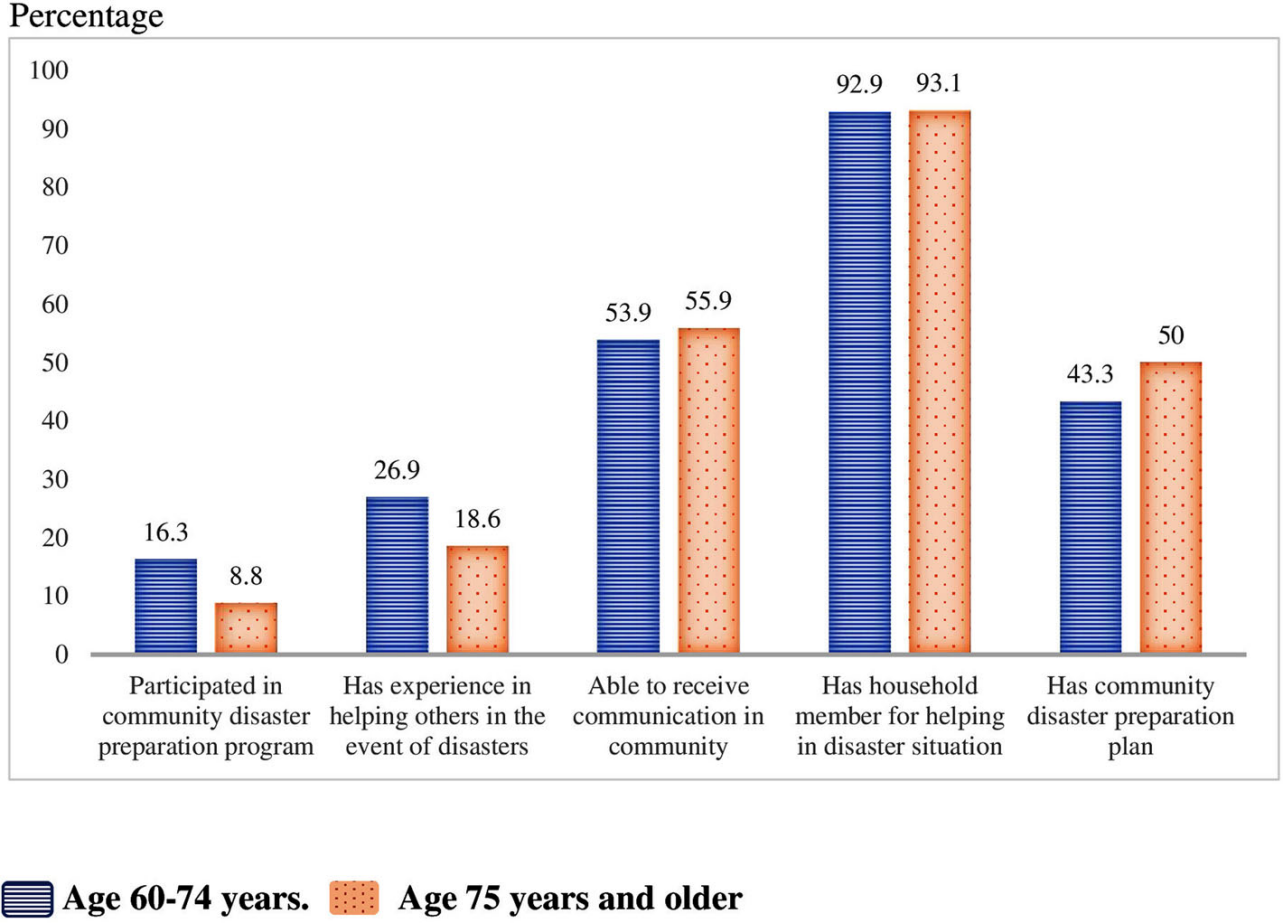
Community and family preparedness indicator (percent as percentage)

## Discussion

This study demonstrated the low knowledge (disaster preparedness indicator) and high behavior responses (participated in disaster preparedness course, knew disaster risk in their community, and had emergency plan for disaster situation) of elderly ED patients in one middle-income country for disaster preparedness. Disaster preparedness guidelines exist in developed countries such as in 2012 the Center for Disease Control and Prevention “Identifying Vulnerable Older Adults and Legal Options for Increasing Their Protection During All-Hazards Emergencies” [20] and in 2013 American College of Emergency Physician “Disaster Planning Toolkit for the Elderly and Special Needs Persons” [21]. Still, there are limitations in the ability to prepare and develop preparedness guidelines to keep older adults from harm or injury, including a lack of consensus on the most effective way to identify and protect older adults in a middle-income country. Our survey may assist in planning for the first step of disaster preparedness among older adults in a middle-income country.

Two- thirds of the study population reported that they had good knowledge of disaster preparedness and more than 80 % reported that a healthcare worker provided knowledge about disasters. Our results are unlike Alrousan TM et al’s study [18] of older adults in the US that reported two thirds had never participated in any disaster preparedness educational program. One reason for our high reported knowledge was that our survey was performed after the Bangkok flood in 2011 [22], which affected almost all Bangkok hospitals. Healthcare providers may have more awareness and enthusiasm to provide community disaster preparedness education.

Most of the elderly ED patients had underlying diseases that required long-term medications; interestingly more than 80% reported that they have a medication supply of 3 weeks. These results may be due to several reasons. First, our country has universal coverage healthcare [23], which covers all medical expenses for the Thai population. Second, the ratio of patients per physician is high which leads physicians to order more months of medications for chronic diseases given the lack of clinic appointment availability.

In terms of specific disaster preparedness questions, only 20 % of elderly ED patients knew specific shelter locations and more than one third did not have an emergency tool kit. These findings were consistent with a study by Morin VM, et al. [2] in the Philippines, which shares a geographic risk for natural disasters, such as flood, as in Thailand. Two-thirds of elderly ED patients did not know the emergency telephone number and telephone for emergency calls; and even fewer in the old older adults group. Half of elderly ED patients reported functional decline and mobility limitations. Impaired physical mobility, diminished sensory awareness, and chronic health conditions make elderly patients vulnerable to disaster situations and inadequate preparation for disasters [24].

In terms of safety issues, most households used natural gas but little is known about how to cut off power. In this situation guidelines suggest a comprehensive emergency preparedness plan for specific needs such as a communication plan, transfer plan and safety issues plan. For example, using simple technology for communication and promoting emergency telephone numbers.

Most of the elderly ED patients in this study reported having family support. With an aging population, the demand for family support or caregivers is crucial. Each family who takes care of elderly persons with chronic diseases such as dementia or stroke should be encouraged to have emergency plans and not rely on one caregiver who may be unable to adequately assist the dependent elderly ED patients [25]. Disaster preparedness for older populations is a global need. Planning should address the issues of general and emergency health requirement for older adults.

This study has several limitations. This was one single center study so results may not be generalizable. Data were collected at specific times and may not reflect future preparedness capacities. Also, this survey focused on the attitudes of patients, the results may not reflect the actual knowledge about disaster preparedness. We did not use an objective scale to assess knowledge such as asking about the contents in emergency toolkit or actual telephone numbers. For the answers to the items in this survey we relied on self-reported answers that may be affected by memory. Not every respondent had experienced disasters so the findings may not reflect the true situation. However, some of the findings may be of value for disaster situations and may aid in planning for the first step of disaster preparedness in a middle-income country.

## Conclusions

While most Thai elderly ED patients reported having a medication supply for disaster situations, many lacked comprehensive plans for a disaster situation. Given the increasing number of older adults, global warming and other disaster risks, we need more public health and prevention planning and programs to improve the quality of preparedness in disaster situations. Future research should focus on preparedness knowledge regarding evacuation, and shelter/residence for older patients.

## Data Availability

Data sharing not applicable to this articles as no datasets were generated or analysed during the current study.

## Abbreviations

ED: Emergency department
EPs: Emergency physicians
IQR: Interquartile range
IRB: Institutional Review Boar
PTSD: Post-traumatic stress disorder
RAs: Research assistants
SPMSQ: Short Portable Mental State Questionnaire
